# Long-term efficacy of fluralaner (Exzolt^®^) in *Gallus gallus domesticus* against epidemiologically relevant triatomines (Hemiptera: Reduviidae: Triatominae): a potential complementary strategy for Chagas disease control

**DOI:** 10.1186/s13071-026-07382-5

**Published:** 2026-04-18

**Authors:** Luanderson Cardoso Pereira, Nathalie de Sena Pereira, Denis Dantas da Silva, Kivia Millana de Sousa, Clarice de Freitas Bezerra, Jéssica Martins Sanches, Lívia Fagundes Viana Bosnic, Luiz Gustavo Rodrigues Oliveira, Carlos Ramon do Nascimento Brito, Nelder Figueiredo Gontijo, Andressa Noronha Barbosa da Silva, Antonia Claudia Jácome da Câmara, Lúcia Maria da Cunha Galvão, Manuela Sales Lima Nascimento, Gabriel L. Hamer, Cleber Galvão, Rita de Cássia Moreira de Souza, Marcos Horácio Pereira, Paulo Marcos Matta Guedes

**Affiliations:** 1 Graduate Program in Parasitology, Federal University of Minas Gerais, Belo Horizonte, Minas Gerais Brazil; 2 Graduate Program in Parasite Biology, Federal University of Rio Grande Do Norte, Natal, Rio Grande do Norte Brazil; 3 Department of Microbiology and Parasitology, Federal University of Rio Grande Do Norte, Natal, Rio Grande do Norte Brazil; 4 Center for Biological and Health Sciences, Federal University of Oeste da Bahia, Barreiras, Bahia Brazil; 5 Department of Clinical and Toxicological Analyses, Federal University of Rio Grande Do Norte, Natal, Rio Grande do Norte Brazil; 6 Department of Parasitology, Federal University of Minas Gerais, Belo Horizonte, Minas Gerais Brazil; 7 Graduate Program in Health Sciences, Federal University of Rio Grande Do Norte, Natal, Rio Grande do Norte Brazil; 8 Department of Entomology, Texas A&M University, College Station, Texas USA; 9 Instituto Oswaldo Cruz–FIOCRUZ, Rio de Janeiro, Rio de Janeiro Brazil; 10 Grupo Triatomíneos, Instituto René-Rachou–FIOCRUZ, Belo Horizonte, Minas Gerais Brazil

**Keywords:** Fluralaner (Exzolt®), Chicken, Chagas disease, *Rhodnius*, *Triatoma*, *Panstrongylus*

## Abstract

**Background:**

Chagas disease control relies primarily on vector control using pyrethroid insecticide sprays with residual action in domestic and peridomestic environments. However, the necessity for repeated applications and the development of pyrethroid resistance in some countries have undermined this strategy. Alternative control tools are needed, and host-targeted systemic insecticides offer an alternative approach by creating toxic blood meals for hematophagous vectors. In peridomestic settings, chickens are major blood meal sources for triatomines. This study evaluated the insecticidal activity of orally administered fluralaner (Exzolt^®^) to chickens against six triatomine species of epidemiological relevance for the transmission of *Trypanosoma cruzi*, the etiological agent of Chagas disease, in Latin America: *Rhodnius prolixus, Triatoma infestans, Triatoma dimidiata, Triatoma brasiliensis, Triatoma pseudomaculata and Panstrongylus megistus*.

**Methods:**

Sixteen non-breeding chickens (*Gallus gallus domesticus*) were randomized by weight into four groups: group 1, untreated control (*n* = 4); group 2, treated with two doses of 0.5 mg/kg fluralaner/Exzolt® (*n* = 4); group 3, treated with two doses of 2.5 mg/kg fluralaner/Exzolt® (*n* = 4); group 4, treated with two doses of 5.0 mg/kg fluralaner/Exzolt® (*n *= 4). To assess fluralaner (Exzolt®) efficacy, chickens were exposed to blood feeding by triatomines at baseline (day 0) and 1, 7, 14, 21, 28, 35, 56 and 77 days post-treatment. Mortality was monitored daily for up to 7 days after each feeding.

**Results:**

Treatment with 0.5, 2.5 and 5.0 mg/kg of Exzolt® resulted in 100% insecticidal activity in triatomines for up to 14, 21 and 28 days post-treatment, respectively. The 2.5 and 5.0 mg/kg doses produced comparable insecticidal activity, both superior than that observed at 0.5 mg/kg.

**Conclusions:**

Oral administration of fluralaner (Exzolt®) to chickens induces 100% insecticidal activity and maintains insecticidal efficacy against multiple triatomine species for up to 28 and 56 days post-treatment, respectively. These findings highlighting the potential of fluralaner as a complementary vector control strategy for Chagas disease in endemic areas.

**Graphical Abstract:**

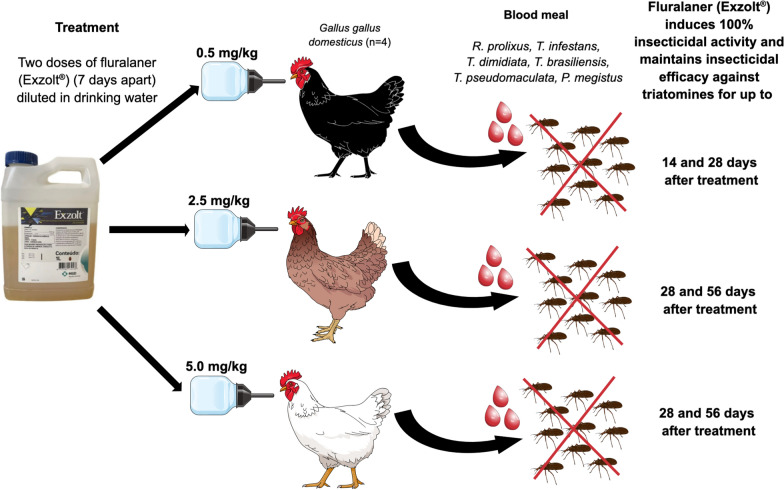

**Supplementary Information:**

The online version contains supplementary material available at 10.1186/s13071-026-07382-5.

## Background

*Trypanosoma cruzi* is the etiological agent of Chagas disease, with an estimated 6–7 million people infected and nearly 100 million at risk worldwide [1]. In endemic areas of the Americas, parasite transmission to humans occurs mainly through biological transmission by triatomine bugs (Hemiptera: Reduviidae: Triatominae), which excrete infectious metacyclic trypomastigotes in their feces, leading to contamination of the host [2]. Parasite transmission can also occur via include oral, congenital and blood transfusion routes [1, 3]. Although 158 triatomine species have been described [4], only a few are directly involved in the transmission of *T. cruzi* to humans, and their epidemiological relevance varies regionally according to abundance, host feeding patterns and domiciliation and intrusion behavior [2]. Based on these factors, *Rhodnius prolixus, Triatoma infestans*, *Triatoma dimidiata*, *Triatoma brasiliensis* and *Panstrongylus megistus* are considered to be the primary vectors of *T. cruzi* in regions of Latin America [5–7]. Also,* T. pseudomaculata* plays a particularly important role in the semi-arid region of Brazil, within the Caatinga biome [8, 9].

The application of pyrethroid insecticides in domestic and peridomestic areas remains the primary strategy for interrupting *T. cruzi* transmission by triatomine vectors. Nevertheless, the residual activity of these compounds in peridomestic environments is often short-lived, typically persisting for only a few weeks due to environmental factors such as wind, rainfall and sunlight [3, 7, 10, 11]. Moreover, the intensive and prolonged use of pyrethroids has driven the emergence of resistant triatomine populations in several Latin American regions [12], thereby compromising the effectiveness of vector control efforts. Therefore, developing novel triatomine control approaches to complement current insecticide-based interventions is crucial to reducing vector populations and minimizing the risk of human infection with *T. cruzi*.

Triatomines are obligate hematophagous insects that require multiple blood meals throughout their life-cycle [13]. Consequently, the use of systemic insecticides administered to domestic vertebrates that serve as major blood sources for triatomines in peridomestic environments represents a promising strategy for vector control [14, 15]. Oral administration of systemic insecticides to vertebrate hosts frequently fed upon by triatomines can render their blood toxic, resulting in insect death through xenointoxication [16]. In peridomestic settings, chicken coops are consistently among the most heavily triatomine-infested habitats [17–20]. Although chickens are not susceptible to *T. cruzi* infection, they play a key role as providers of blood meals, supporting triatomine populations in close proximity to humans [21–25]. Consequently, chickens represent strategic targets for intervention, as their treatment could limit triatomine populations and reduce the risk of human infection. Several active ingredients, including deltamethrin, permethrin, cypermethrin, imidacloprid, fipronil, ivermectin and isoxazolines, are widely used in animals for their endoparasiticidal and/or ectoparasiticidal activity [15].

Fluralaner (Exzolt® for poultry; Bravecto® for dogs; Merck Animal Health [MSD Animal Health outside of the USA and Canada], Merck & Co., Inc., Rahway, NJ, USA) is a systemic insecticide of the isoxazoline class and a potent inhibitor of arthropod γ-aminobutyric acid (GABA) and glutamate-gated chloride channels (GluCls), leading to hyperexcitability and, consequently, death [26–28]. Previous studies have shown that administration of fluralaner (Bravecto®) to chickens induces insecticidal activity against triatomines (*Triatoma gerstaeckeri* and *T. infestans*) for up to 14 days post-treatment [29, 30]. Our group further demonstrated that Exzolt® administered at 0.5 mg/kg elicited insecticidal activity lasting to 28 days against *T. infestans*, *R. prolixus*, *T. brasiliensis* and *T. pseudomaculata* [31]. However, the insecticidal activity of fluralaner against *T. dimidiata* and *P. megistus* had not yet been evaluated. Therefore, the aim of this study was to assess the insecticidal activity of fluralaner/Exzolt® administered to chickens against six epidemiologically relevant triatomine species (*T. infestans, T. brasiliensis, T. pseudomaculata, T. dimidiata, R. prolixus* and *P. megistus*), using the manufacturer-recommended dose (0.5 mg/kg) as well as higher doses (2.5 and 5.0 mg/kg) to explore whether increased dosing extends the duration of activity.

## Methods

### Insects

The specimens of *T. infestans, T. brasiliensis, T. pseudomaculata, T. dimidiata, R. prolixus* and *P. megistus* used in this experiment (*n* = 1440 third [N3]-, fourth [N4]- and fifth [N5]-instar nymphs) were obtained from colonies maintained at the Laboratory of Immunoparasitology (LIP), Federal University of Rio Grande do Norte (UFRN). Colonies of *T. pseudomaculata* and *T. brasiliensis* originated from insects captured in the municipalities of Serra Negra do Norte and Caraúbas, state of Rio Grande do Norte, Brazil. *Triatoma infestans* (Espinosa and Bambuí, Minas Gerais, Brazil), *P. megistus* (Jaboticatubas, Minas Gerais, Brazil) and *R. prolixus* (Honduras) were obtained from colonies maintained at the Instituto René Rachou (FIOCRUZ-MG, Brazil), whereas *Triatoma dimidiata* (Mexico) was obtained from colonies at the Instituto Oswaldo Cruz (FIOCRUZ-RJ, Brazil). The triatomines in these colonies were maintained through weekly blood-feeding on mice, rats or chickens, and kept in an insectary in glass cages covered with mesh, under controlled conditions: darkness, 60% relative humidity and 28 °C. For the present experiment, after molting, N3, N4 and N5 nymphs were fed on healthy mice and subsequently starved for approximately 20 days before the chicken blood-feeding experiment was conducted. All triatomines used in the experiment were free of *T. cruzi* infection.

### Chickens

A total of 16 adult, non-breeding chickens (*Gallus gallus domesticus*), aged 1–3 years, were sourced from a private farm in Parnamirim, Rio Grande do Norte, Brazil. The animals were maintained in groups of four in separate cages with ad libitum access to feed and water. No insecticide treatments had been applied to the birds or their housing environment for at least 12 months prior to the study, nor were any treatments administered during the experimental period.

### Drug

Chickens were treated with fluralaner (10 mg/ml) (Exzolt®), which was diluted in the drinking water provided to the birds during a 24-h period. Fluralaner was administered at three doses: 0.5 mg/kg, 2.5 mg/kg and 5.0 mg/kg, each given in two administrations spaced 7 days apart. The volume of Exzolt® required for each group was calculated based on the average body weight of the birds and their water intake on the day preceding treatment.

### Assessment of adverse effects in chickens

To evaluate potential adverse effects of orally administered fluralaner (Exzolt®), chickens were monitored for clinical and behavioral changes throughout the experimental period. Clinical assessments included general activity, appetite, water intake, dyspnea, ataxia, tremors, convulsions, paralysis, diarrhea, mucous membrane coloration and signs of dehydration. Clinical observations were recorded daily during the first 7 days post-treatment and once per week thereafter until the end of the study. Body weight was recorded on 7 days prior to treatment (day − 7) and on days 1, 7, 14, 21, 28, 35, 56 and 77 post-treatment.

### Study design

**Fig. 1:**
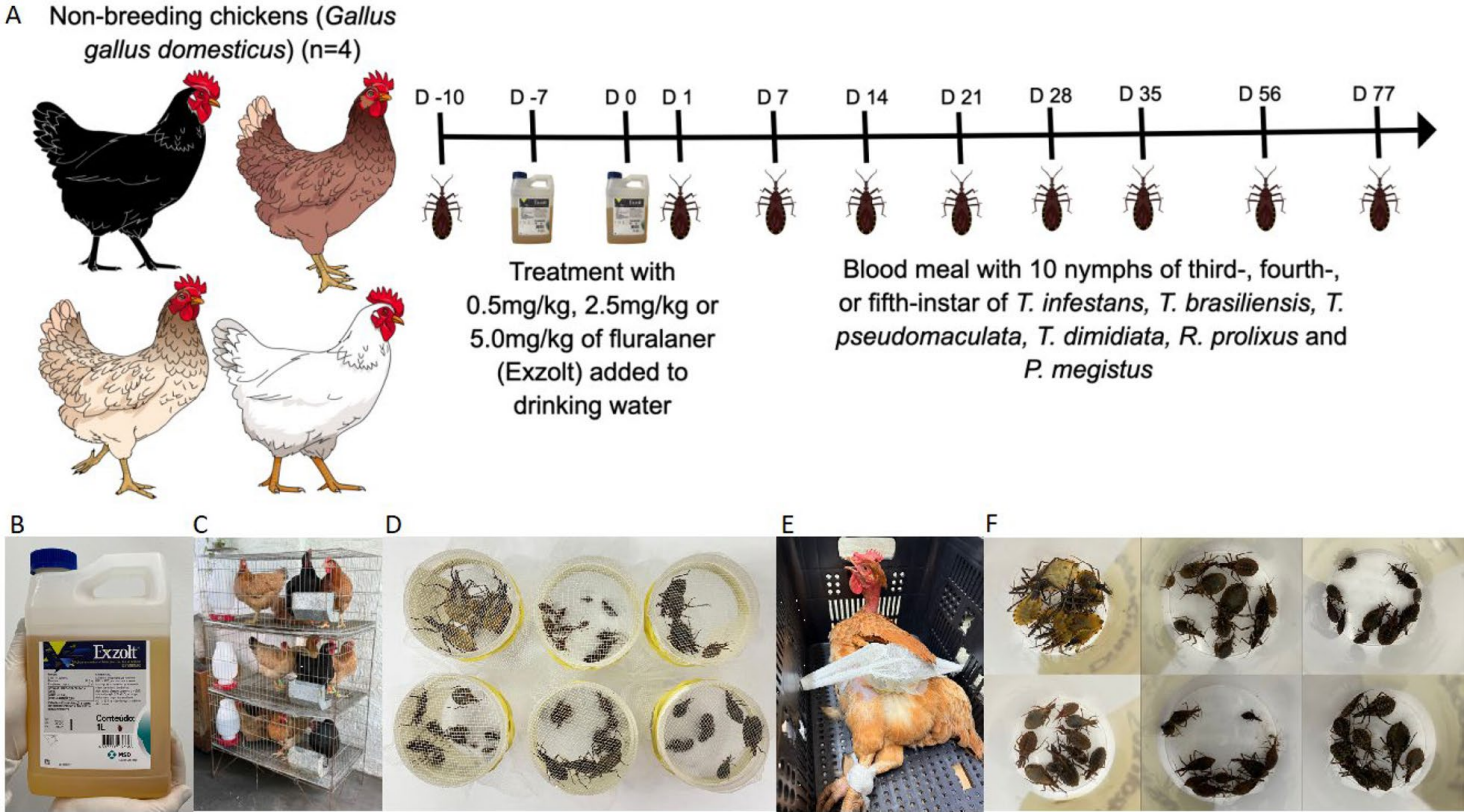
Bioassay workflow for evaluating the insecticidal activity of fluralaner (Exzolt®) against triatomines. Chickens were treated with two doses (**A**, **B**) of 0.5, 2.5 or 5.0 mg/kg of fluralaner (Exzolt®), administered in drinking water (**C**) for 24 h. Insecticidal activity was then assessed at 1, 7, 14, 21, 28, 35, 56 and 77 days post-treatment (**D–F**) Plastic containers (50 ml) covered with fine mesh and containing ten third−, fourth− or fifth-instar nymphs of *Rhodnius prolixus*, *Triatoma infestans*, *Triatoma dimidiata*, *Triatoma brasiliensis*, *Triatoma pseudomaculata* and *Panstrongylus megistus* were held for 30 min against the lateral body region below the wings (**D**). For blood-feeding, each chicken was gently restrained with gauze and placed laterally inside black plastic boxes (**E**) to facilitate triatomine access. After feeding, unfed insects were removed, and engorged nymphs were maintained in an insectarium (dark conditions, 50% relative humidity, 28 °C) for 7 days to assess mortality (**F**)

The 16 mixed-breed chickens (*Gallus gallus domesticus*) were randomized into four groups: group 1, untreated/control (*n* = 4); group 2, treated with two doses of 0.5 mg/kg fluralaner/Exzolt® (*n* = 4); group 3, treated with two doses of 2.5 mg/kg fluralaner/Exzolt® (*n* = 4); and group 4, treated with two doses of 5.0 mg/kg fluralaner/Exzolt® (*n* = 4), with the aim of evaluating insecticidal activity against triatomines. Control and treated chickens were subjected to blood-feeding at baseline and at 1, 7, 14, 21, 28, 35, 56 and 77 days post-treatment by N3, N4 and N5 nymphs of each triatomine species. For blood-feeding, chickens were positioned laterally in a plastic box and gently immobilized with gauze to minimize movement and ensure proper insect access. Containers holding 10 nymphs, covered with a fine mesh lid, were placed under the wings and left in place for 30 min to allow feeding. After feeding, insects were transferred to an insectary (60% humidity, 28 °C, darkness) at the UFRN Immunoparasitology Laboratory and monitored daily for 7 days to assess mortality (Fig. 1). Feeding efficiency was determined by measuring nymph body weight before and after feeding, expressed as the fold increase in body weight.

### Statistical analysis

The insecticidal activity of fluralaner (Exzolt®) against triatomines was evaluated by comparing the mean mortality rates of insects that fed on treated chickens, as no deaths occurred among insects fed on untreated controls. Differences in mortality between treated and control groups, as well as within treated groups across time, were analyzed using a generalized linear mixed model (GLMM) for repeated measures, which included fluralaner treatment, feeding efficiency, time, insect species and their interactions as fixed effects. The model employed an autoregressive AR(1) covariance structure, and pairwise comparisons were corrected using the Bonferroni adjustment. Feeding efficiency before and after blood meals, as well as across post-feeding intervals, was analyzed using the Kruskal–Wallis test followed by Dunn’s multiple comparison test. Spearman’s test was used to correlate the mortality with the volume of blood ingested by triatomines. Statistical analyses were conducted using SPSS software version 20.0 (SPSS IBM Corp., Armonk, NY, USA) and GraphPad Prism version 10 (GraphPad Software, San Diego, CA, USA). A significance threshold of *p* < 0.05 was adopted, and all graphs were generated in GraphPad Prism 10.

## Results

### Treatment of chickens with two doses of 0.5, 2.5 and 5.0 mg/kg fluralaner (Exzolt®) does not cause adverse effects in chickens and does not affect the feeding efficiency of triatomines

**Fig. 2:**
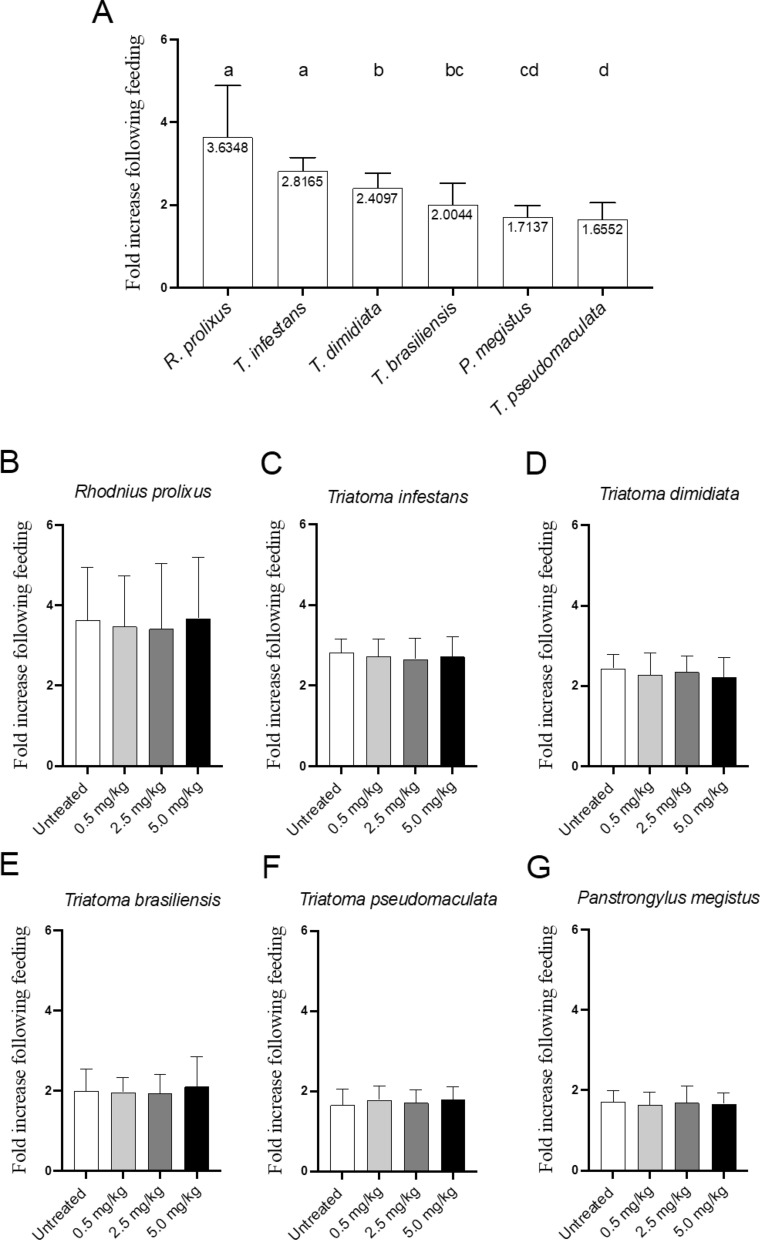
Treatment of chickens with fluralaner (Exzolt®) does not impair triatomine feeding success. Feeding efficiency was first evaluated on untreated chickens (**A**) to identify species with higher feeding propensity.** B–G** Feeding success of *Rhodnius prolixus* (**B**), *Triatoma infestans* (**C**), *Triatoma dimidiata* (**D**), *Triatoma brasiliensis* (**E**), *Triatoma pseudomaculata* (**F**) and *Panstrongylus megistus* (**G**) was assessed after feeding on control (untreated) and fluralaner-treated chickens receiving two doses of fluralaner (Exzolt®) at 0.5, 2.5 or 5.0 mg/kg. Feeding success was determined by weighing nymphs before and after the blood-feeding and calculating the fold increase in body weight. Assessments were conducted before treatment and at 1, 7, 14, 21, 28, 35, 56 and 77 days post-treatment. Different lowercase letters above the bars indicate statistically significant differences at *p* < 0.05

No adverse effects were observed in chickens treated with two doses of 0.5, 2.5 or 5.0 mg/kg fluralaner (Exzolt®) throughout the 77-day observation period. The fold increase in nymphal body weight after blood-feeding was used as the biomarker for feeding success; on untreated chickens, the mean (± standard deviation [SD]) fold increase was 3.63 ± 1.78 for *R. prolixus* (*n *= 320), 2.81 ± 0.82 for *T. infestans* (*n* = 320), 2.40 ± 0.84 for *T. dimidiata* (*n* = 320), 2.0 ± 0.67 for *T. brasiliensis* (*n* = 320), 1.65 ± 0.71 for *T. pseudomaculata* (*n* = 320) and 1.71 ± 0.61 for *P. megistus* (*n* = 320) (Fig. 2a). Overall, the results indicate that *R. prolixus* and *T. infestans* exhibited the highest feeding success, followed by *T. dimidiata* and *T. brasiliensis*, which showed intermediate values, whereas the lowest feeding success on chickens was recorded for *P. megistus* and *T. pseudomaculata* (Fig. 2a).

Assessment of feeding success showed no difference (*p* = 1.000) in the engorgement level of triatomines that fed on untreated control chickens compared with those that fed on birds treated with two doses of 0.5, 2.5 or 5.0 mg/kg fluralaner, suggesting that fluralaner administration does not impact the insects’ ability to feed **(**Fig. 2b–g**).** The mean (± SD) fold increase in body weight of nymphs across the periods of 1, 7, 14, 21, 28, 35, 56 and 77 days post-treatment in chickens treated with 0.5, 2.5 or 5.0 mg/kg fluralaner (Exzolt®) and in untreated chickens was 2.72 ± 1.18, 2.65 ± 0.92, 2.71 ± 0.99 and 2.81 ± 0.82, respectively, for *T. infestans*; 1.96 ± 1.03, 1.94 ± 0.67, 2.11 ± 0.68 and 2.0 ± 0.67, respectively, for *T. brasiliensis*; 1.78 ± 0.71, 1.71 ± 0.49, 1.79 ± 0.49 and 1.65 ± 0.71, respectively, for *T. pseudomaculata*; 2.27 ± 0.97, 2.34 ± 0.94, 2.22 ± 0.97 and 2.40 ± 0.84, respectively, for *T. dimidiata*; 1.63 ± 0.65, 1.68 ± 0.66, 1.66 ± 0.62 and 1.71 ± 0.61, respectively, for *P. megistus*; and 3.47 ± 1.88, 3.41 ± 2.06, 3.67 ± 1.9 and 3.63 ± 1.78, respectively, for *R. prolixus*. Together, these findings confirm that treatment of chickens with fluralaner (Exzolt®) does not alter triatomine blood-feeding performance and does not induce adverse effects in the birds.

### Mortality was influenced by engorgement level of triatomines

**Fig. 3:**
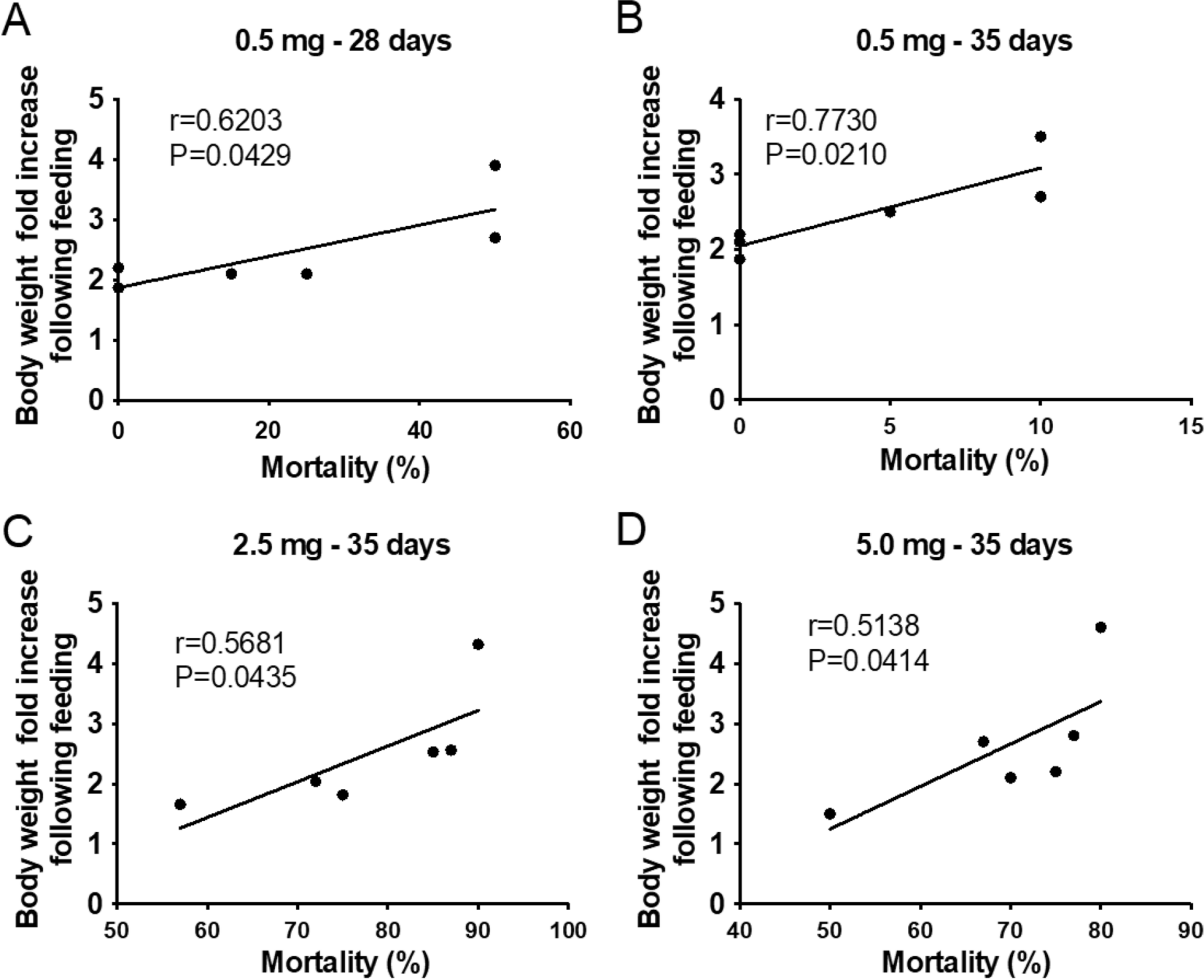
Triatomine mortality was positively correlated with engorgement level after feeding on fluralaner (Exzolt®)-treated chickens. Correlations between mortality and blood intake (determined by fold increase in body weight) were observed in chickens treated with 0.5 mg/kg at days 28 (**A**) and 35 (**B**), and at day 35 in chickens treated with 2.5 mg/kg (**C**) and 5.0 mg/kg (**D**). Individual data points represent the mean mortality and mean fold increase in body weight for each triatomine species, and the line represents the linear regression for each comparison. Spearman’s rank correlation test was performed, and statistical significance was set at* p* < 0.05

The GLMM analysis, independent of time after treatment and dose, showed that triatomine mortality was significantly influenced by engorgement level in *R. prolixus* (*p* = 0.032), *T. infestans* (*p* = 0.037), *T. dimidiata* (p < 0.001), *T. brasiliensis* (*p* = 0.043), *T. pseudomaculata* (*p* = 0.045) and *P. megistus* (*p* = 0.040). When the volume of blood ingested in relation to mortality, stratified by exposure time, was analyzed, we observed that mortality was influenced by the degree of engorgement at the 0.5 mg/kg dose on days 28 and 35, and at the 2.5 and 5.0 mg/kg doses on day 35 after treatment. Notably, these periods correspond to the onset of declining insecticidal activity. Correlation analysis between triatomine mortality and feeding efficiency (measured as fold increase in body weight) demonstrated a positive correlation at the 0.5 mg/kg dose on days 28 (*p* < 0.0429;* r* = 0.6203) (Fig. 3a) and 35 (*p* = 0.0210;* r *= 0.7730) **(**Fig. 3b) after treatment, and at the 2.5 mg/kg (*p* = 0.0435;* r* = 0.5681) (Fig. 3c) and 5.0 mg/kg (*p* = 0.0414;* r* = 0.5138) (Fig. 3d) doses on day 35 after treatment, regardless of the triatomine species evaluated. In contrast, no significant correlation between mortality and feeding efficiency was detected at the other time points assessed.

### Oral administration of fluralaner to chickens results in prolonged insecticidal activity against triatomines

**Fig. 4:**
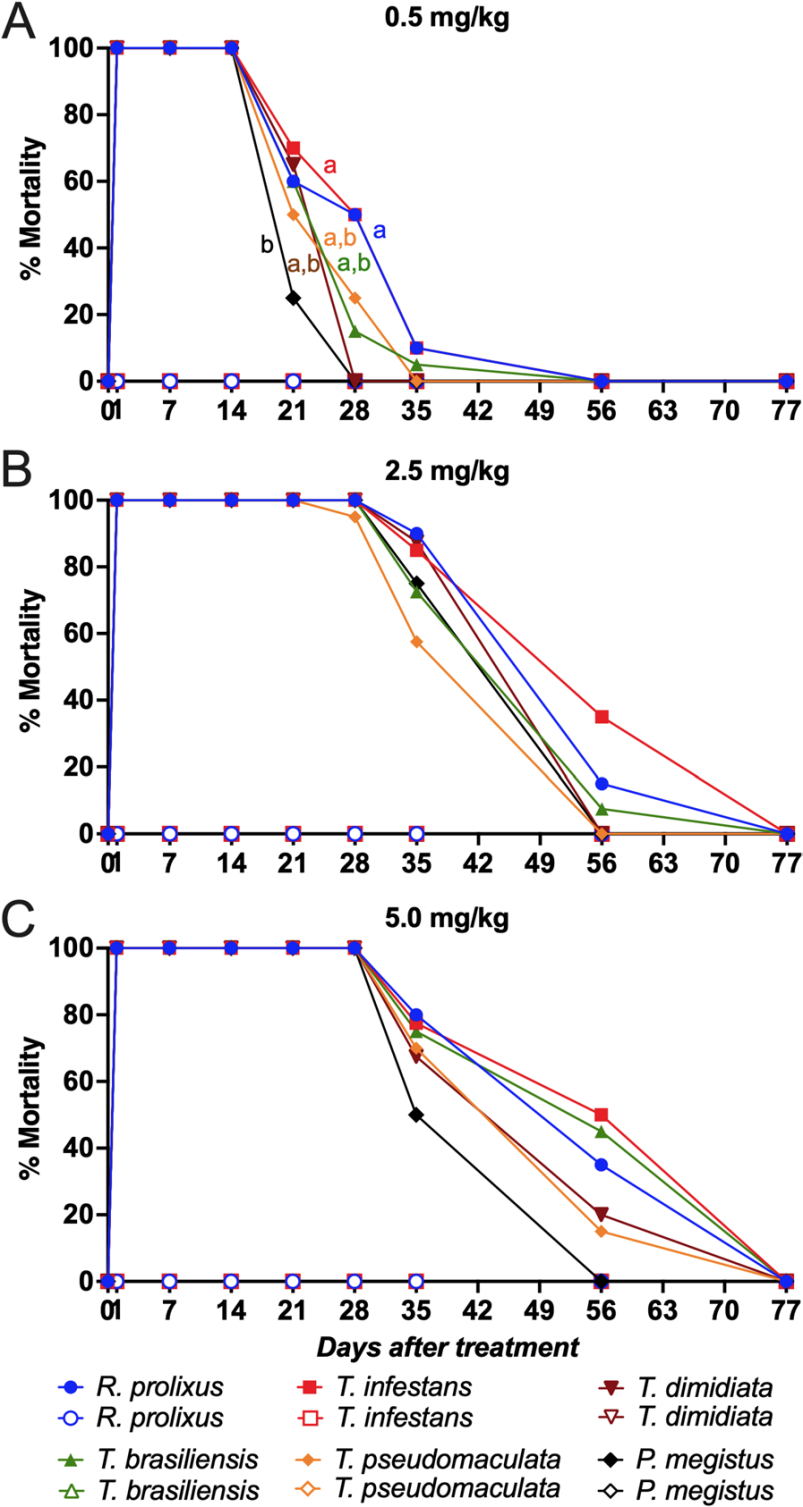
Insecticidal activity of fluralaner (Exzolt®) at 2.5 and 5.0 mg/kg against six triatomine species. Chickens were treated with two doses of 0.5 mg/kg (**A**), 2.5 mg/kg (**B**) or 5.0 mg/kg (**C**) fluralaner (Exzolt®), and mortality (%) of *Rhodnius prolixus*, *Triatoma infestans*, *Triatoma dimidiata*, *Triatoma brasiliensis*, *Triatoma pseudomaculata* and *Panstrongylus megistus* was assessed before treatment and 1, 7, 14, 21, 28, 35, 56 and 77 days after treatment. At each time point, ten third-, fourth- and fifth-instar nymphs fed on each chicken. Mortality values represent means, and a generalized linear mixed model (GLMM) was used for comparisons among groups. Mortality curves marked with different lowercase letters indicate statistically significant differences at *p* < 0.05. Filled symbols represent insects that fed on fluralaner-treated chickens, and open symbols represent insects fed on untreated control chickens

The assessment of insecticidal activity was based on cumulative triatomine mortality recorded on the 7th day after blood-feeding. No mortality was observed in insects that fed on untreated control chickens (Fig. 4).

Across all species, two oral doses of fluralaner (0.5 mg/kg) induced rapid and pronounced insecticidal activity, with 100% mortality at 1, 7 and 14 days post-treatment (Fig. 4; Additional file 1: Table S1). In the group treated with 0.5 mg/kg of fluralaner, mortality remained significantly higher up to 28 days post-treatment for *R. prolixus* (GLMM,* F* = 64.25,* df* = 29.15, *p* < 0.002) and *T. infestans* (GLMM,* F * = 58.57,* df* = 30.44, *p* < 0.010), and up to 21 days for *T. brasiliensis* (GLMM,* F* = 92.91,* df* = 33.63, *p* < 0.001), *T. pseudomaculata* (GLMM,* F* = 174.76,* df* = 42.30, *p* < 0.001) and *T. dimidiata* (GLMM,* F* = 165.72,* df *= 38.33, *p* < 0.001), when compared to pre-treatment mortality (0%). Insecticidal activity against *P. megistus* (GLMM,* F* = 136.40,* df* = 44.58, *p* < 0.001) was observed up to 14 days post-treatment with the 0.5 mg/kg dose (Fig. 4; Additional file 1: Table S1). No insecticidal activity was detected at 35, 56 or 77 days post-treatment for any triatomine species (Fig. 4; Additional file 1: Table S1).

A dose of 2.5 mg/kg of fluralaner provided sustained and near-complete insecticidal activity across all triatomine species. Mortality reached 100% for *R. prolixus*, *T. infestans*, *T. dimidiata*, *T. brasiliensis* and *P. megistus* up to 28 days post-treatment, while mortality reached 95% for *T. pseudomaculata* at 28 days post-treatment (Fig. 4; Additional file 2: Table S2). Mortality remained significantly higher in the group treated with 2.5 mg/kg of fluralaner up to 56 days post-treatment for *T. infestans* (GLMM,* F* = 58.57,* df* = 30.44, *p* = 0.045), and up to 35 days for *R. prolixus* (GLMM,* F* = 64.25,* df* = 29.15, *p* < 0.001), *T. dimidiata* (GLMM,* F* = 165.72,* df* = 38.33, *p* < 0.001), *T. brasiliensis* (GLMM,* F* = 92.91,* df* = 33.63, *p* < 0.001), *T. pseudomaculata* (GLMM,* F* = 174.76,* df* = 42.30, *p* < 0.001) and *P. megistus* (GLMM,* F* = 136.40,* df *= 44.58, *p* < 0.001), when compared to pre-treatment mortality (0%). No insecticidal activity was detected at 77 days post-treatment, when mortality returned to control levels for all species (Fig. 4; Additional file 2: Table S2).

The 5.0 mg/kg fluralaner regimen yielded complete insecticidal activity for up to 28 days post-treatment against all triatomine species evaluated (Fig. 4; Additional file 3: Table S3). Mortality remained significantly higher in the group treated with 5.0 mg/kg of fluralaner up to 56 days post-treatment for *R. prolixus* (GLMM,* F* = 64.25,* df* = 29.15, *p* = 0.049), *T. infestans* (GLMM,* F* = 58.57,* df* = 30.44, *p* = 0.010) and *T. brasiliensis* (GLMM,* F *= 92.91,* df *= 33.63, *p* = 0.004), and up to 35 days for *T. dimidiata* (GLMM,* F* = 165.72,* df* = 38.33, *p* < 0.001), *T. pseudomaculata* (GLMM,* F* = 174.76,* df* = 42.30, *p* < 0.001) and *P. megistus* (GLMM,* F* = 136.40,* df *= 44.58, *p* < 0.001), when compared to pre-treatment mortality (0%). By 77 days post-treatment, insecticidal activity was no longer detectable, with mortality returning to baseline for all species (Fig. 4; Additional file 2: Table S2, Additional file 3: Table S3).

**Fig. 5:**
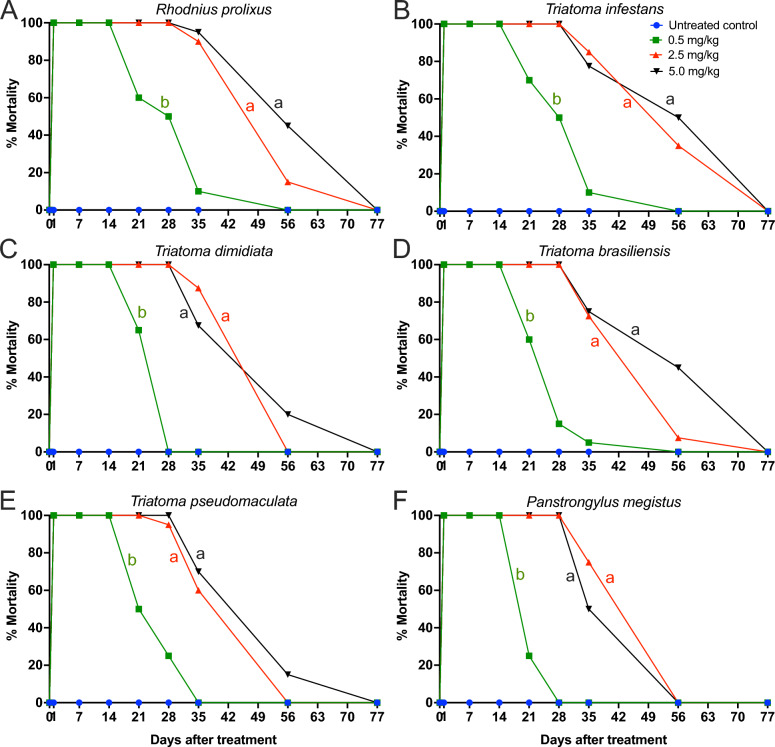
Higher insecticidal efficacy of 2.5 mg/kg and 5.0 mg/kg of fluralaner (Exzolt®), compared with the 0.5 mg/kg dose. Chickens were treated with two doses of 0.5, 2.5 or 5.0 mg/kg of fluralaner (Exzolt®). Mortality (%) of *Rhodnius prolixus* (**A**)**,** *Triatoma infestans* (**B**)**,** *Triatoma dimidiate* (**C**), *Triatoma brasiliensis* (**D**)**,** *Triatoma pseudomaculata* (**E**), and *Panstrongylus megistus* (**F**) was assessed before treatment and at 1, 7, 14, 21, 28, 35, 56 and 77 days post-treatment. Ten third-, fourth- and fifth-instar nymphs were fed on each chicken at each time point. Data are expressed as mean values, and mortality among treatment groups was compared using a generalized linear mixed model (GLMM). Mortality curves with different lowercase letters indicate statistically significant differences at *p* < 0.05

The doses of 2.5 and 5.0 mg/kg exhibited similar insecticidal activity, with the insecticidal activity of both doses superior to that of the 0.5 mg/kg dose (Fig. 5; Additional file: Table S1; Additional file: Table S2, Additional file 3: Table S3). Treatment with 2.5 and 5.0 mg/kg fluralaner showed higher insecticidal efficacy against *R. prolixus* (*p* = 0.006 and *p* = 0.004, respectively) (Fig. 5a), *T. infestans* (*p* = 0.011 and *p* = 0.006, respectively) (Fig. 5b), *T. dimidiata* (*p* < 0.001 and *p* < 0.001, respectively) (Fig. 5c), *T. brasiliensis* (*p* < 0.001 and *p* < 0.001, respectively) (Fig. 5d), *T. pseudomaculata* (*p* < 0.001 and *p* < 0.001, respectively) (Fig. 5e) and *P. megistus* (*p* < 0.001 and *p* < 0.001, respectively) (Fig. 5f), compared to treatment with 0.5 mg/kg dose.

## Discussion

The results of the present study demonstrate that treating chickens with 2.5 or 5.0 mg/kg of fluralaner (Exzolt®) induces insecticidal activity against triatomines for up to 56 days post-treatment and that, compared with the manufacturer’s recommended dose of 0.5 mg/kg, extends the period of 100% insecticidal activity from 14 to 28 days.

In the present study, we evaluated the insecticidal activity of fluralaner (Exzolt®) against six triatomine species of high epidemiological relevance for the transmission of *T. cruzi* to humans in Latin America, using three doses (0.5, 2.5 and 5.0 mg/kg) of fluralaner (Exzolt®) administered to chickens. In a previous study, our group demonstrated the insecticidal activity of fluralaner (Exzolt®) for up to 28 days following the administration of two doses of 0.5 mg/kg in chickens [31]. Here, we evaluated higher dosing regimens, with the aim of prolonging systemic insecticidal activity. The administration of fluralaner (Exzolt®) at 0.5, 2.5 and 5.0 mg/kg, respectively, produced no adverse effects in chickens throughout the experimental period, indicating its favorable safety profile. Similarly, previous studies reported the absence of side effects in chickens treated with one or two doses of 0.5 mg/kg of fluralaner (Exzolt®) [31] or fluralaner (Bravecto®) [29, 32]. In addition, the use of two doses of 1.0, 1.5 or 2.5 mg/kg of fluralaner (Exzolt^®^) did not result in adverse reactions, further supporting its good safety profile [33, 34]. Indeed, in no adverse reactions were observed in previous studies in which chickens were treated at up to fivefold the recommended dose for threefold the recommended treatment duration, thereby demonstrating a substantial safety margin—up to a cumulative dose equivalent to 15-fold the recommended one (7.5 mg/kg body weight) in healthy adult laying hens [33, 35].

Subsequently, we evaluated whether fluralaner (Exzolt®) treatment affected the feeding success of triatomines. The administration of two doses of 0.5, 2.5 or 5.0 mg/kg of fluralaner to chickens did not alter the feeding success of triatomines compared with insects fed on untreated control chickens. Previous studies have also shown that the treatment of chickens with 0.5 mg/kg of fluralaner (Exzolt®) or fluralaner (ravecto®) does not interfere with the feeding success or engorgement level of *T. infestans*, *T. brasiliensis*, *T. pseudomaculata*, *R. prolixus* and *T. gerstaeckeri* [21, 31]. Consistent with these results, the treatment of chickens with fluralaner has also been shown not to induce any repellent effect on *Culex quinquefasciatus* [36]. Numerous investigations have shown that certain insecticides, particularly pyrethroids, have repellent effects on phlebotomine sand flies [37–39]. However, our results demonstrate that fluralaner (Exzolt®) does not exert any repellent effect on *T. infestans, T. brasiliensis, T. pseudomaculata, T. dimidiata, R. prolixus* and *P. megistus.* Thus, the insecticidal activity observed is attributable solely to systemic exposure to fluralaner and not to any deterrent effect on host-seeking or feeding behavior.

Treatments with 2.5 and 5.0 mg/kg, respectively, exhibited similar insecticidal activity, both of which were superior to that observed at the 0.5 mg/kg dose (manufacturer’s recommended dosage), for *T. infestans, T. brasiliensis, T. pseudomaculata, T. dimidiata, R. prolixus* and *P. megistus*. Previously published data demonstrate that increasing doses (0.25, 0.5, and 1.0 mg/kg) of fluralaner (Exzolt®) led to a corresponding increase in insecticidal efficacy against *Ornithonyssus sylviarum* infestations in laying hens [27]. Treatment with 0.5, 2.5 and 5.0 mg/kg of fluralaner (Exzolt^®^) resulted in 100% insecticidal activity against triatomines for up to 14, 21, and 28 days, respectively. Moreover, our results demonstrate that the treatment of chickens with 0.5 mg/kg, 2.5 and 5.0 mg/kg of fluralaner induced insecticidal activity lasting up to 28, 56, and 56 days post-treatment, respectively. Previous data show that treatment of chickens with two doses of 0.5 mg/kg of fluralaner (Exzolt®) induced 100% insecticidal activity against *R. prolixus*, *T. infestans* and *T. brasiliensis* up to 14 days post-treatment, with sustained activity up to 28 days post-treatment [31]. The treatment of chickens with two doses of fluralaner (Bravecto®), the formulation intended for dogs, resulted in insecticidal activity against *T. gerstaeckeri* and *T. infestans* for up to 14 days post-treatment [29, 30]. However, chicken treatments with Bravecto® may be substantially less effective than those with Exzolt®, as Exzolt® induces higher plasma concentrations of fluralaner than those achieved with Bravecto® [28, 29, 32, 35].

The insecticidal efficacy of fluralaner (Exzolt®) at the 0.5 mg/kg dose varied depending on the species of triatomine. Higher insecticidal activity against *Rhodnius prolixus* and *T. infestans* compared to *P. megistus* was recorded. A higher insecticidal efficacy of the 0.5 mg/kg dose of fluralaner (Exzolt®) administered to chickens has also been reported previously for *R. prolixus* and *T. infestans* compared to *T. brasiliensis* and *T. pseudomaculata* [31]. In contrast, treatment with 2.5 and 5.0 mg/kg of fluralaner (Exzolt®) resulted in similar and consistently high insecticidal activity across all triatomine species evaluated. The observed variation in insecticidal activity among species at the lower dose may reflect intrinsic genetic differences between species, as well as variation in the ratio of blood meal volume to body weight among the tested insects. In the present study, we demonstrated that the amount of blood ingested influences the mortality of triatomines that fed on chickens treated with fluralaner (Exzolt®). The positive correlation analysis between blood intake and mortality was observed during post-treatment periods when a decline in insect mortality was detected, likely reflecting a reduction in plasma fluralaner concentrations. Evidence from the literature also indicates increased mortality in bed bugs and triatomines that fully fed on chickens treated with fluralaner (Bravecto®), compared with partially engorged insects [29, 32]. Another factor that may influence susceptibility to fluralaner is the number of GABA- and GluCl- channels present in each species. A plausible hypothesis is that insects with fewer of these receptors might require a lower concentration of fluralaner to achieve inhibition. However, this notion remains speculative and warrants experimental investigation. Nevertheless, all triatomine species tested were susceptible to the insecticidal action of fluralaner, and no evidence of resistance was detected.

In rural communities, poultry farming represents an important source of protein through the consumption of eggs and meat. According to the Exzolt® label [35], the administration of fluralaner (Exzolt®) at a dose of 0.5 mg/kg to chickens necessitates that the meat and viscera not be consumed for 14 days post-treatment (14-withdrawal period), whereas no withdrawal period is required for eggs. The maximum residue limits of fluralaner in meat, viscera and eggs for human consumption are 0.65, 0.65 and 1.3 mg/g, respectively [40–42]. However, a recent study demonstrated that the administration of fluralaner (Exzolt®) at 0.5 mg/kg by the intravenous route in healthy Shaver laying hens resulted in a withdrawal period of 7 days for eggs [40]. Data on fluralaner quantification in eggs and meat remain scarce in the literature. Withdrawal periods may vary among different chicken breeds. Indeed, variations in drug metabolism and residue depletion in eggs among chicken breeds have also been reported for other pharmaceutical compounds [43, 44]. Therefore, the length of the withdrawal periods for eggs, meat and viscera following administration of the recommended dose by the manufacturer (0.5 mg/kg)—and higher doses—still needs to be more thoroughly investigated before field application.

The practical recommendations for field application of fluralaner (Exzolt®) remain to be clearly defined. In this study, we used three doses: the manufacturer’s recommended dose (0.5 mg/kg); a high dose (5.0 mg/kg) that falls within the maximum dosage evaluated by the manufacturer and considered safe for administration in chickens (15.0 mg/kg divided into 2 doses of 7.5 mg/kg administered at a 7-day interval) [35]; and an intermediate dose between these two (2.5 mg/kg). We included the 5.0 mg/kg dose based on the hypothesis that a higher dose would result in a longer period of insecticidal activity. However, we found no statistically significant difference in insecticidal activity between the 2.5 and 5.0 mg/kg doses. Further studies evaluating doses of 1.0, 1.5 and 2.0 mg/kg are warranted to determine the dose that achieves maximal insecticidal activity while using the lowest concentration of fluralaner, with the aim of field implementation. As a cost reference, 1 l of fluralaner (10 mg/ml; Exzolt®) is sufficient to fully treat approximately 5000 chickens weighing 2 kg each, using two administrations of the 0.5 mg/kg dose. In Brazil, this volume is priced at around US$ 1000 (February 2026), resulting in an estimated per-animal treatment cost of approximately US$ 0.2.

From an operational perspective, the strategic treatment of chickens in infested communities shows strong potential to reduce triatomine populations and domestic reservoirs, decrease reinfestation rates after insecticide spraying and help manage pyrethroid-resistant populations. The use of commercially available formulations is advantageous, as it facilitates regulatory approval and programmatic implementation. Moreover, water-administered Exzolt does not cause stress to hens and minimizes workers’ exposure to the pyrethroids used in spraying. In addition, the application logistics are more favorable than spraying, which may lead to greater treatment compliance. The convenient administration of the ready-to-use solution via drinking water reduces the workload compared to spraying, which requires removal of birds and/or eggs, frequent reapplications (especially in peridomestic areas), quarantines and the use of extensive personal protective equipment. However, important challenges must be considered before field application. These include careful evaluation of withdrawal periods and residue levels in meat and eggs, particularly at doses higher than those recommended on the label, in order to avoid human exposure. The cost of treatment, the logistical feasibility of repeated administrations and community acceptance should also be considered. Therefore, field-based cost-effectiveness analyses and pharmacovigilance studies are essential to guide its safe and sustainable incorporation into vector control programs.

## Conclusions

Taken together, these results indicate that treatment with two doses of 2.5 or 5.0 mg/kg of fluralaner exhibited similar insecticidal activity, both of which were superior to the 0.5 mg/kg dosage, against all triatomine species evaluated. Oral administration of 2.5 and 5.0 mg/kg fluralaner (Exzolt®) to chickens induces 100% insecticidal activity and maintains insecticidal efficacy against epidemiologically relevant triatomine species for up to 28 and 56 days, respectively. Overall, the results support the use of fluralaner (Exzolt®) as a promising complementary vector control strategy for Chagas disease in endemic areas. Future studies should evaluate the efficacy of fluralaner under field conditions and its integration with Chagas vector control programs.

## Supplementary Information

**Additional file 1: Table S1.** Triatomines mortality after two doses of fl uralaner (0.5 mg/kg) administered in chickens. Statistical analysis was performed using generalized linear mixed model (GLMM) for repeated measures comparing triatomine mortality before treatment with the different periods evaluated after treatment.

**Additional file 2: Table S2.** Triatomines mortality after two doses of fl uralaner (2.5 mg/kg) administered in chickens. Statistical analysis was performed using generalized linear mixed model (GLMM) for repeated measures comparing triatomine mortality before treatment with the different periods evaluated after treatment.

**Additional file 3: Table S3. ** Triatomines mortality after two doses of fl uralaner (5.0 mg/kg) administered in chickens. Statistical analysis was performed using generalized linear mixed model (GLMM) for repeated measures comparing triatomine mortality before treatment with the different periods evaluated after treatment.

## Data Availability

Data supporting the conclusions of the present study are included within the article.
