# Supplementary material for: Long-term efficacy of fluralaner (Exzolt®) in Gallus gallus domesticus against epidemiologically relevant triatomines (Hemiptera: Reduviidae: Triatominae): a potential complementary strategy for Chagas disease control
Source: Parasit Vectors. 2026 Apr 18;19:233. doi: 10.1186/s13071-026-07382-5 (PMC13220561; doi:10.1186/s13071-026-07382-5)
Supplement: Supplementary file 3 — Additional file 3: Table S3. Triatomines mortality after two doses of fl uralaner (5.0 mg/kg) administered in chickens. Statistical analysis was performed using generalized linear mixed model (GLMM) for repeated measures comparing triatomine mortality before treatment with the different periods evaluated after treatment. [file 13071_2026_7382_MOESM3_ESM.docx]

**Supplementary Table 3. Triatomines mortality after two doses of fluralaner (5.0 mg/kg) administered in chickens.** Statistical analysis was performed using generalized linear mixed model (GLMM) for repeated measures comparing triatomine mortality before treatment with the different periods evaluated after treatment.

| **Triatomine species/days after treatment** | **1** | **7** | **14** | **21** | **28** | **35** | **56** | **77** |
| --- | --- | --- | --- | --- | --- | --- | --- | --- |
| *Rhodnius prolixus (%)*  *(p value)* | 100  (< 0.001) | 100  (< 0.001) | 100  (< 0.001) | 100  (< 0.001) | 100  (< 0.001) | 80.0  (< 0.001) | 35  (= 0.049) | 0  (= 1.000) |
| *Triatoma infestans* | 100  (< 0.001) | 100  (< 0.001) | 100  (< 0.001) | 100  (< 0.001) | 100  (< 0.001) | 77.5  (< 0.001) | 50.0  (= 0.010) | 0  (= 1.000) |
| *Triatoma dimidiata* | 100  (< 0.001) | 100  (< 0.001) | 100  (< 0.001) | 100  (< 0.001) | 100  (< 0.001) | 67.5  (< 0.001) | 20.0  (= 1.000) | 0  (= 1.000) |
| *Triatoma brasiliensis* | 100  (< 0.001) | 100  (< 0.001) | 100  (< 0.001) | 100  (< 0.001) | 100  (< 0.001) | 75.0  (< 0.001) | 45.0  (= 0.004) | 0  (= 1.000) |
| *Triatoma pseudomaculata* | 100  (< 0.001) | 100  (< 0.001) | 100  (< 0.001) | 100  (< 0.001) | 100  (< 0.001) | 70.0  (< 0.001) | 15.0  (= 1.000) | 0  (= 1.000) |
| *Panstrongylus megistus* | 100  (< 0.001) | 100  (< 0.001) | 100  (< 0.001) | 100  (< 0.001) | 100  (< 0.001) | 50.0  (< 0.001) | 0  (= 1.000) | 0  (= 1.000) |
